# Concurrent cisplatin or cetuximab with radiotherapy for HPV-positive oropharyngeal cancer: Medical resource use, costs, and quality-adjusted survival from the De-ESCALaTE HPV trial

**DOI:** 10.1016/j.ejca.2019.10.025

**Published:** 2020-01

**Authors:** David A. Jones, Pankaj Mistry, Matthew Dalby, Tessa Fulton-Lieuw, Anthony H. Kong, Janet Dunn, Hisham M. Mehanna, Alastair M. Gray

**Affiliations:** a Health Economics Research Centre, Nuffield Department of Population Health, University of Oxford, Oxford, UK; b Warwick Clinical Trials Unit, University of Warwick, Coventry, UK; c Institute of Head and Neck Studies and Education, Institute of Cancer and Genomic Sciences, College of Medical and Dental Sciences, University of Birmingham, Birmingham, UK

**Keywords:** Oropharyngeal squamous cell carcinoma, Human papillomavirus, Chemoradiotherapy, Cisplatin, Cetuximab, Overall survival, Recurrence, Resource use, Costs, Quality of life

## Abstract

**Background:**

The De-ESCALaTE HPV trial confirmed the dominance of cisplatin over cetuximab for tumour control in patients with human papillomavirus (HPV)-positive oropharyngeal squamous cell carcinoma (OPSCC). Here, we present the analysis of health-related quality of life (HRQoL), resource use, and health care costs in the trial, as well as complete 2-year survival and recurrence.

**Materials and methods:**

Resource use and HRQoL data were collected at intervals from the baseline to 24 months post treatment (PT). Health care costs were estimated using UK-based unit costs. Missing data were imputed. Differences in mean EQ-5D-5L utility index and adjusted cumulative quality-adjusted life years (QALYs) were compared using the Wilcoxon signed-rank test and linear regression, respectively. Mean resource usage and costs were compared through two-sample t-tests.

**Results:**

334 patients were randomised to cisplatin (n = 166) or cetuximab (n = 168). Two-year overall survival (97·5% vs 90·0%, HR: 3.268 [95% CI 1·451 to 7·359], p = 0·0251) and recurrence rates (6·4% vs 16·0%, HR: 2·67 [1·38 to 5·15]; p = 0·0024) favoured cisplatin. No significant differences in EQ-5D-5L utility scores were detected at any time point. At 24 months PT, mean difference was 0·107 QALYs in favour of cisplatin (95% CI: 0·186 to 0·029, p = 0·007) driven by the mortality difference. Health care costs were similar across all categories except the procurement cost and delivery of the systemic agent, with cetuximab significantly more expensive than cisplatin (£7779 [P < 0.001]). Consequently, total costs at 24 months PT averaged £13517 (SE: £345) per patient for cisplatin and £21064 (SE: £400) for cetuximab (mean difference £7547 [95% CI: £6512 to £8582]).

**Conclusions:**

Cisplatin chemoradiotherapy provided more QALYs and was less costly than cetuximab bioradiotherapy, remaining standard of care for nonsurgical treatment of HPV-positive OPSCC.

## Highlights

• Cisplatin was superior to cetuximab for tumour control.
• Quality-adjusted survival was significantly higher with cisplatin.
• Patients had similar medical resource utilisation in both arms.
• Cetuximab was more costly due to higher procurement cost.
• Chemoradiotherapy-related costs accounted for half of total per-patient costs.

## 1 Introduction

The incidence of oropharyngeal squamous cell carcinoma (OPSCC) is rising in many developed countries, driven principally by increasing infection rates of oncogenic human papillomavirus (HPV) [1,2]. HPV-positive OPSCC represents a distinct disease entity to its HPV-negative counterpart. While the latter is typically induced by excessive smoking and/or alcohol consumption, HPV-positive patients are often younger and healthier, characterised by favourable prognosis with half the risk of death [3].

Nevertheless, current treatment practices do not differentiate between disease types, and are associated with acute and late toxicities. This morbidity is of particular concern for HPV-positive patients given the favourable long-term survival rates and young age of diagnosis, leading many patients to live with poor health-related quality of life (HRQoL) over extended periods. Management of treatment-related sequelae also imposes considerable additional costs on the health care system, as well as privately on the individual.

Consequently, there has been a refocusing of the therapeutic paradigm for HPV-positive OPSCC towards de-escalation, which ideally reduces treatment-related toxicities without compromising tumour control. Cetuximab, a monoclonal antibody against epidermal growth factor receptor, is one of the first treatments under investigation for de-escalation [4]. The potential clinical benefit of cetuximab for head and neck squamous cell carcinoma was first demonstrated in a randomised controlled trial of radiotherapy versus radiotherapy plus cetuximab [5,6]. This led to the investigation of its comparative effectiveness versus standard care cisplatin–based chemoradiotherapy for HPV-positive OPSCC in the De-ESCALaTE HPV (ISRCTN33522080) international open-label randomised controlled phase III trial [7].

De-ESCALaTE HPV recently reported expedited results of their comparison of radiotherapy plus concurrent cisplatin or cetuximab, with the primary outcome of difference in severe (grade 3–5) toxicity events. Compared with the standard cisplatin regimen, cetuximab showed no benefit in terms of reduced toxicity, but significant detriment in terms of tumour control [7]. These results were in line with those from the multicentre NRG Oncology RTOG 1016 noninferiority trial [8]. The cisplatin regimen did result, however, in significantly more serious adverse events (SAEs) [7].

A prespecified secondary objective of De-ESCALaTE HPV was to compare medical resource use, costs, and HRQoL in the two study arms, and we now report this analysis. Although the survival results were unfavourable to cetuximab, the trial does provide reliable information on medical resource use, related costs, and HRQoL as measured by the generic multiattribute EQ-5D-5L utility instrument after standard care cisplatin and radiotherapy in this population. With many other de-escalation treatments strategies under investigation, such data are vital to help evaluate these strategies against current standard care. We also report completed estimates from the trial of 2-year overall survival and time to recurrence.

## 2 Materials and methods

### 2.1 Study

Full details of the De-ESCALaTE HPV trial can be found in the previously published results paper [7]. Briefly, eligible patients were aged 18 years or older with low-risk HPV-positive advanced OPSCC, defined according to the Ang classification [3] as nonsmokers or smokers with a lifetime history of <10 pack-years, with positive p16 immunohistochemistry. Patients were recruited from treatment centres in Ireland (n = 1), the Netherlands (n = 1), and the UK (n = 30), and randomly assigned (1:1) through a minimisation algorithm including centre, tumour stage (TNM7: T1–T2 vs T3–T4), nodal stage (N0–1 vs N2–3), radiotherapy site (unilateral; bilateral), and planned gastrostomy insertion before treatment. Therapy consisted of radiotherapy (70 Gy in 35 fractions), with either intravenous cisplatin (100 mg/m2 on days 1, 22, and 43 of radiotherapy) or intravenous cetuximab (400 mg/m^2^ initial dose followed by seven weekly infusions of 250 mg/m^2^). Patients were followed up for a minimum of two years with monthly examinations at the clinic in the first year, and every two months in the second year, in line with normal clinical practice to detect recurrence early.

### 2.2 Health care resource use and quality of life data collection, and attribution of costs

Throughout the trial, data on resource use were collected by means of case report forms (CRFs) and resource use questionnaires (RUQs) which were adapted from RUQs used in several previous trials and found to have high completion and low error rates [[9], [10], [11]]. During treatment, CRFs collected information on the administration of radiotherapy and radiosensitising agent, including whether radiotherapy was completed, number of chemo/biotherapy cycles received, cycle dose, and for the cisplatin arm any switches to carboplatin. Ancillary items given during chemo/biotherapy such as hydration and anti-emetics were obtained from prespecified centre regimen documents. Follow-up CRFs recorded details of all hospital admissions, as well as contacts with the consultant, and any imaging performed. The RUQs were given at the baseline, end of treatment (on average two months after baseline), and 6, 12, and 24 months post treatment (PT), with patients asked to recall their use of health care services over the intervening period. Each questionnaire contained items on hospital-based care including inpatient stays, day centre and outpatient clinic visits, accident and emergency contacts, and convalescent and nursing home stays. It also contained items on primary and community care activities such as GP, nurse, social worker, and therapist visits. There were overlaps between the data collected in the CRFs and RUQs concerning hospitalisations, imaging, and consultant visits, and a summary of the approach taken to reconcile these can be found in the online Appendix.

Unit costs associated with resource usage were obtained from UK-based sources including the Department of Health and Social Care's drugs and pharmaceutical electronic market information tool, the British National Formulary, National Health Service reference costs, and the Unit Costs of Health and Social Care [[12], [13], [14], [15]]. Details of unit costs and their sources are provided in the online Appendix.

HRQoL was assessed using one of the most widely used generic preference-based measures, the EQ-5D-5L questionnaire, which was administered at the baseline, end of treatment, and 3, 6, 12, and 24 months PT [16]. The EQ-5D-5L questionnaire covers five health domains: mobility, self-care, usual activities, pain/discomfort, and anxiety/depression. Patients then identify their current health status for each domain as one of five levels: no problems, slight problems, moderate problems, severe problems, and extreme, leading to 3125 possible health states. Each health state can be attributed a utility index score using a valuation set (also known as tariff), which results in a preference-based score ranging from <0 (states worse than dead) to 1 (full health), with dead anchored at 0.

### 2.3 Statistical analysis

The primary analysis was performed on all randomised patients under the intention-to-treat principle. A secondary analysis was performed on the per-protocol population, excluding patients who withdrew or who had major protocol violations as assessed by the independent trial monitoring team. Descriptive statistics of the trial population by treatment arm were derived, including means, medians, standard deviations, and interquartile ranges as appropriate. 2-year overall survival and time to recurrence were estimated, for the intention-to-treat population only, using the approach previously outlined [7].

Mean resource usage, costs, and their corresponding standard errors (SE) by category were summarised for each trial arm. Mean differences and 95% confidence intervals (CIs) were calculated and compared through two-sample t-tests.

EQ-5D utility index scores at each time point were derived by mapping EQ-5D-5L responses to the EQ-5D-3L tariff using the scoring algorithm of Van Hout *et al.*, the method currently recommended by the National Institute for Health and Care Excellence [17,18]. Differences in the distribution of EQ-5D-5L responses across the domains at each time point were compared using Fisher's exact test. Differences in mean EQ-5D utility index scores at each time point between the two arms were compared using the Wilcoxon signed-rank test. Quality-adjusted life years (QALYs) for each patient were calculated using area under the curve after linear interpolation between time points, with adjustment for date of death where relevant. Finally, cumulative mean QALYs over the follow-up period were estimated with adjustment for baseline index score, gender, and number of comorbidities at randomisation. Neither resource use, costs, nor QALYs were discounted.

Where patients had partially completed the RUQs, it was assumed that resource use items left blank had not been used within the relevant follow-up period. Following best practice for the conduct of economic evaluations alongside clinical trials, missing data from partially completed EQ-5D-5L and fully incomplete RUQs and EQ-5D-5L questionnaires were imputed through multiple imputation by chained equations under a missing-at-random assumption [19,20]. Here, an imputation model is specified for each incomplete variable. Missing entries are imputed in an iterative process, cycling repeatedly along the imputation models to converge at a value for each missing entry, thereby avoiding dependence on the order in which the variables are imputed. Missing values were imputed separately by treatment arm, at the item level for resource use and at the tariff level for EQ-5D-5L. All missing variables were imputed using predictive mean matching to allow for discrete target variables and provide robustness against non-normality [21]. Predictive mean matching ‘borrows’ values from the set of observed data points with regression-predicted values closest to the predicted value of the missing entry [22].

Covariates for each missing variable imputation model included all other resource use and/or EQ-5D-5L variables across all time points, as well as age, gender, TNM stage, ECOG performance status, number of comorbidities, and planned gastrostomy insertion before treatment. A total of 20 sets of imputed values were obtained. Rubin's rule was used to generate combined estimates of means and SEs across MI data sets where appropriate [23]. Complete case analysis restricted to the set of patients who had fully completed questionnaires at each time point was also performed for comparison, the results of which are available in the online Appendix. Analysis of resource use, costs, and HRQoL was performed using R version 3.5.1 [24]. Survival and recurrence rates were analysed in STATA version 15.1 [25].

## 3 Results

### 3.1 Study

A total of 334 patients were randomised between November 2012 and October 2016, 166 to cisplatin and 168 to cetuximab, of whom 159 and 162, respectively, made up the per-protocol population. Baseline characteristics for patients in each arm of the trial are presented in Table 1. The groups were well balanced with respect to demographic and clinical characteristics including disease/symptom severity. In the following we report the intention-to-treat results. Per-protocol results can be found in the online Appendix.

**Table 1:** Baseline characteristics of patients.

Variable	Cisplatin (N = 166)	Cetuximab (N = 168)	Total (N = 334)
Age			
Mean (SD)	57·54 (7·84)	57·46 (8·25)	57·50 (8·04)
Median (IQR)	57·00 (10·10)	57·84 (12·30)	57·37 (10·93)
Gender			
Male	132 (79·5%)	134 (79·8%)	266 (79·6%)
Female	34 (20·5%)	34 (20·2%)	68 (20·4%)
Tumour stage (TNM 7)			
T1-T2	109 (65·7%)	107 (63·7%)	216 (64·7%)
T3-T4	57 (34·3%)	61 (36·3%)	118 (35·3%)
T4 only	32 (19·3%)	24 (14·3%)	56 (16·8%)
Nodal stage (TNM 7)			
N0–N1	40 (24·1%)	41 (24·4%)	81 (24·3%)
N2–N3	126 (75·9%)	127 (75·6%)	253 (75·7%)
N3 only	1 (0·6%)	1 (0·6%)	2 (0·6%)
Primary subsite (N = 329)			
Base of tongue	54 (32·9%)	58 (35·2%)	112 (34·0%)
Tonsil	107 (65·2%)	104 (63·0%)	211 (64·1%)
Other	3 (1·8%)	3 (1·8%)	6 (1·8%)
ECOG performance status (N = 328)			
0	142 (86·6%)	149 (90·9%)	291 (88·7%)
1	22 (13·4%)	15 (9·1%)	37 (11·3%)
Ever smoked? (N = 329)			
No	90 (54·9%)	85 (51·5%)	175 (53·2%)
Yes	74 (45·1%)	80 (48·5%)	154 (46·8%)
Planned PEG use before treatment			
No	57 (34·3%)	58 (34·5%)	115 (34·4%)
Yes	109 (65·7%)	110 (65·5%)	219 (65·6%)

### 3.2 Overall survival and time to recurrence

**Fig. 1:**
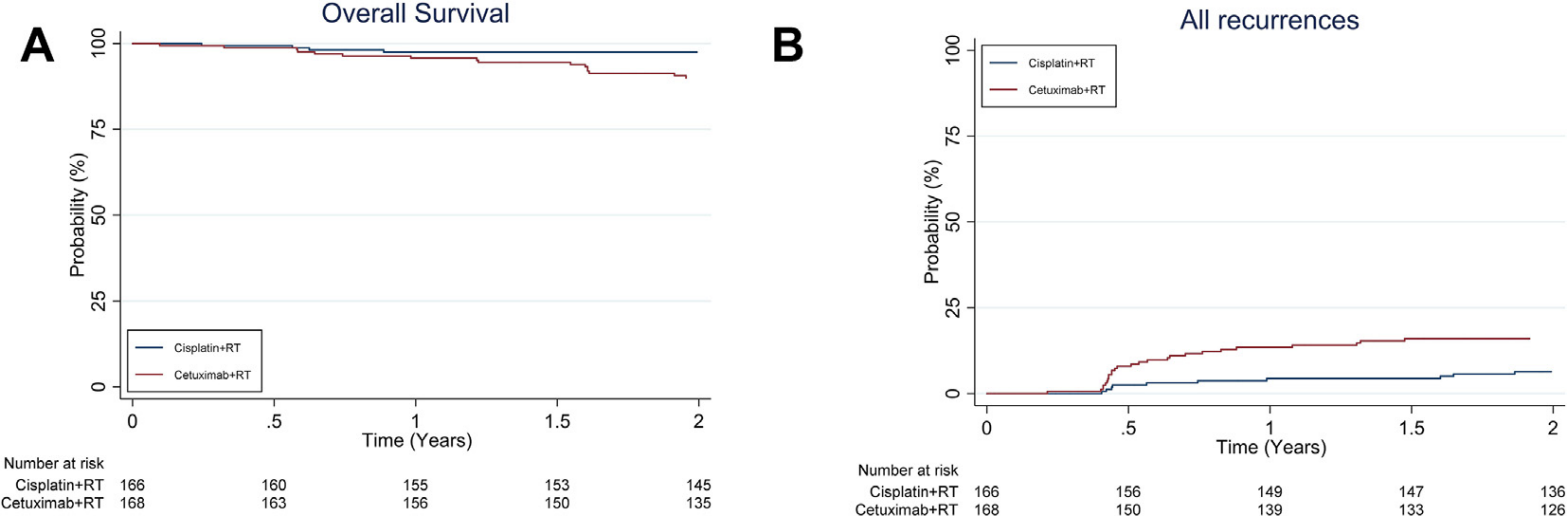
a: 2-year overall survival.b: 2-year time to recurrence.

Results from the recently published expedited results paper showed no benefit from cetuximab in terms of reduced overall severe and all-grade toxicity, and a significant reduction in 2-year overall survival and recurrence [7]. The results of the updated intention-to-treat analysis, with 2-year follow-up for all patients, again showed a significant difference between cisplatin and cetuximab in 2-year overall survival (97·5% vs 90·0%, HR: 3.268 [95% CI 1·451 to 7·359], p = 0·0251; Fig. 1a) and in the 2-year recurrence rate (6·4% vs 16·0%, HR: 2·67 [1·38 to 5·15]; p = 0·0024; Fig. 1b), in favour of cisplatin.

### 3.3 Medical resource use and costs

Mean total resource use and costs over the trial follow-up from the imputed data sets are presented in Table 2. Patients in the cisplatin arm received on average 2·33 (SD: 0·70) cycles, including nine patients who switched to carboplatin, while patients in the cetuximab arm received 7·42 (SD 1·42). The greater number of cycles, as specified in the protocol, and unit cost of cetuximab relative to cisplatin led to a mean difference in total treatment costs per patient of £7779 (95% CI: £7377 to £8182) between the two arms. We found no statistically significant differences in the mean number of hospital inpatient days, day case/outpatient visits, accident and emergency visits, or primary and community care contacts between the two trial arms. Neither was there any difference in associated mean costs for these categories. Total costs after 24 months PT were £13517 (SE: £345, equating to €14135 [SE: €361] using 2018 purchasing price parities [26]) in the cisplatin group and £21064 (SE: £400, €22027 [SE: €418]) in the cetuximab group. Treatment with cetuximab therefore significantly increased total cost per patient by on average £7547 (95% CI: £6512 to £8582, €7892 [95% CI: €6810 to €8974]).

**Table 2:** Medical resource use and costs.

Variable	Cisplatin (n = 166) mean (SE)	Cetuximab (n = 168) Mean (SE)	Mean difference (95% CI)	P-value
Medical resource use				
Treatment cycles	2·33 (SD: 0·70)	7·42 (SD: 1·42)		
Hospital inpatient stays (total days)	10·083 (1·081)	8·458 (1·078)	−1·624 (−4·62 to 1·371)	0·287
Hospital day/outpatient visits	15·311 (1·079)	15·523 (1·323)	0·211 (−3·223 to 3·645)	0·903
Accident and emergency visits	0·395 (0·062)	0·556 (0·084)	0·161 (−0·048 to 0·37)	0·131
Primary and community care contacts	24·802 (2·499)	24·916 (2·189)	0·113 (−6·365 to 6·592)	0·973
Direct medical costs (£)				
Treatment^a^	7142·40 (90·94)	14921·86 (182·30)	7779·47 (7377·24 to 8181·70)	0·000
Hospital inpatient stays	2846·73 (236·42)	2553·18 (243·05)	−293·54 (−959·18 to 372·09)	0·386
Hospital day/outpatient visits	2485·66 (141·55)	2571·33 (175·29)	85·67 (−347·78 to 519·12)	0·697
Accident and emergency visits	63·23 (9·94)	88·95 (13·48)	25·72 (−7·72 to 59·17)	0·131
Primary and community care contacts	972·37 (105·58)	928·55 (85·26)	−43·83 (−309·38 to 221·73)	0·745
Total	13516·79 (345·43)	21063·88 (399·61)	7547·08 (6512·22 to 8581·95)	0·000

a Including study drugs, other medications received during the cycle, delivery costs, and radiotherapy.

### 3.4 Health-related quality of life

There was little difference in the distribution of EQ-5D-5L responses across the domains (Appendix Table 6). Baseline mobility was somewhat worse in the cetuximab group with more patients reporting slight problems rather than no problems. A similar outcome for self-care at 3 months PT was found, although in favour of cetuximab. There were statistically significant differences in the pain/discomfort domain at the end of treatment, although with no clear monotonic trend, and at 3 months PT, with a distributional shift towards worse domain scores in the cetuximab arm.

**Fig. 2:**
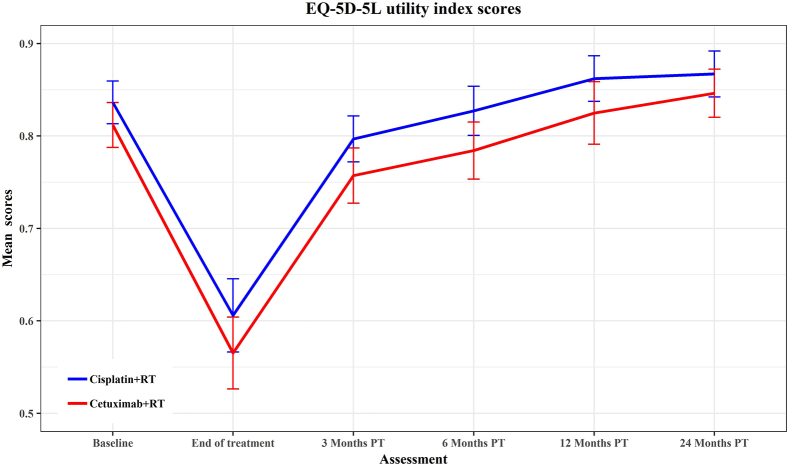
Mean EQ-5D-5L utility index scores.

The EQ-5D utility index score profile across time (Table 3; Fig. 2) showed substantially lower mean HRQoL levels at the end of treatment in both study arms than at the baseline, which then recovered to at least baseline levels by 12 months PT. No significant differences between arms were detected at any time point, although there was a non-negligible difference at the baseline suggesting the need for adjustment in the QALYs analysis.

**Table 3:** Unadjusted mean reported EQ-5D utility index scores.

Time point	Cisplatin		Cetuximab		Utility Difference	p-value (Mann–Whitney U test)
	No· Complete	Mean (SD)	No· Complete	Mean (SD)		
Baseline	155	0·836 (0·147)	152	0·812 (0·153)	0·024	0·080
End of treatment	122	0·606 (0·223)	138	0·565 (0·231)	0·041	0·187
3 months post treatment	130	0·797 (0·145)	130	0·757 (0·173)	0·040	0·084
6 months post treatment	128	0·827 (0·153)	125	0·784 (0·176)	0·043	0·078
12 months post treatment	129	0·862 (0·144)	126	0·825 (0·194)	0·037	0·202
24 months post treatment	120	0·867 (0·139)	118	0·846 (0·144)	0·021	0·131

**Fig. 3:**
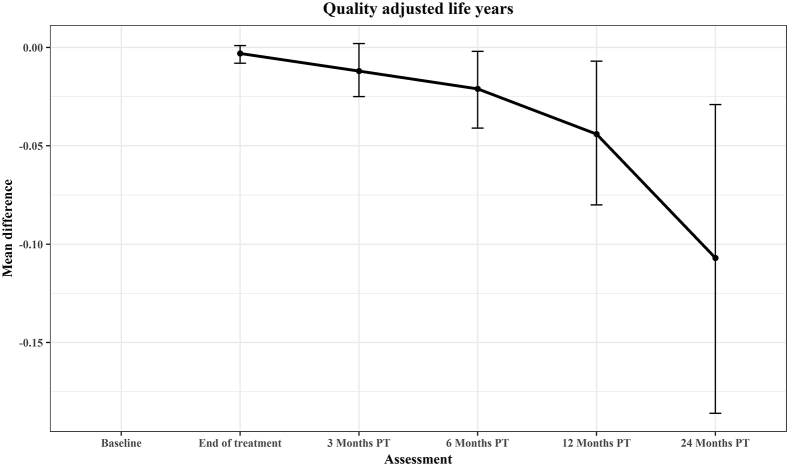
Mean difference in cumulative QALYs. QALYs, quality-adjusted life years.

Table 4 reports cumulative mean QALYs from the baseline to 24 months PT, unadjusted (4a) and then adjusted for baseline values (4b; Fig. 3). The unadjusted results suggest a widening difference over time favouring cisplatin, with a mean cumulative difference after 24 months PT of 0·128 QALYs in favour of cisplatin (95% CI 0·212 to 0·044, p = 0·003). Once adjusted for the baseline EQ-5D-5L utility index score, gender, and comorbidities, significant differences did not emerge until the 6 months PT follow-up point and the mean cumulative difference at 24 months was slightly lower than the unadjusted result at 0·107 QALYs in favour of cisplatin (95% CI: 0·186 to 0·029, p = 0·007). These results were driven primarily by the greater number of deaths in the cetuximab arm.

**Table 4:** Unadjusted and adjusted cumulative mean quality-adjusted life years (QALYs) from baseline.

Time point	Cisplatin		Cetuximab		Mean difference (95% CI)	P-value t-test
	No· Dead	Mean (SE)	No· Dead	Mean (SE)		
**4a: Unadjusted**						
End of treatment	0	0·120 (0·002)	1	0·114 (0·002)	−0·006 (−0·012 to 0·000)	0·058
3 months post treatment	1	0·294 (0·005)	2	0·278 (0·005)	−0·016 (−0·031 to −0·001)	0·031
6 months post treatment	3	0·494 (0·008)	5	0·466 (0·008)	−0·028 (−0·05 to −0·007)	0·011
12 months post treatment	4	0·904 (0·014)	7	0·849 (0·015)	−0·055 (−0·095 to −0·015)	0·007
24 months post treatment	4	1·740 (0·027)	17	1·612 (0·033)	−0·128 (−0·212 to −0·044)	0·003
**4b: Adjusted**						
End of treatment					−0·003 (−0·008 to 0·001)	0·168
3 months post treatment					−0·012 (−0·025 to 0·002)	0·083
6 months post treatment					−0·021 (−0·041 to −0·002)	0·030
12 months post treatment					−0·044 (−0·080 to −0·007)	0·020
24 months post treatment					−0·107 (−0·186 to −0·029)	0·007

## 4 Discussion

Expedited results of the De-ESCALaTE HPV trial demonstrated the superiority of cisplatin over cetuximab for tumour control in HPV-positive OPSCC patients. Our completed 2-year overall survival and 2-year time-to-recurrence analyses confirm the earlier findings. Furthermore, our analysis of the comparative HRQoL and health care costs confers additional support for the superiority of cisplatin. Replacement of cisplatin with cetuximab greatly increased the cost of treatment while providing no statistically significant reduction in medical resource usage or their associated costs. Although, patient-reported HRQoL as measured through the EQ-5D utility index score was similar at each time point among questionnaires returned, quality-adjusted survival was significantly lower in the cetuximab arm due to the greater number of deaths. As such, cisplatin-based chemoradiotherapy should continue to be considered the standard of care in this setting.

The earlier results reported significantly higher number of SAEs in the cisplatin arm compared with the cetuximab arm, mainly due to the increased need for hospital admission [7]. However, despite this, we found no significant difference in the number of inpatient hospital days or outpatient visits and their respective related costs. This suggests that the SAEs may have been less severe, each requiring shorter hospital stays on average. Our results also highlight the drivers of resource utilisation and costs associated with the disease. Of note, the cost of chemo/biotherapy and radiotherapy was the single largest component, accounting for over half of total costs per patient in both trial arms. Finally, our results demonstrate not only the very high rate of survival using cisplatin-based chemoradiotherapy in low-risk HPV-positive OPSCC patients, but also the good HRQoL profile over time, with EQ-5D utility index scores showing that on average patients quickly rebound to the baseline HRQoL values after the end of treatment, and with 24 month PT scores surpassing those at the baseline.

Together, these findings suggest a high bar for other de-escalation strategies, especially those with anticipated higher treatment-specific costs and fewer or less-severe toxicities. Although the unexpected inferiority of cetuximab combined with its higher cost precluded the need for formal cost-effectiveness in this trial, the results also demonstrate the importance of embedding health economic components and analysis into future trials investigating de-escalation strategies, to further aid clinical decision-making.

## Funding

This study was supported by Cancer Research UK [grant number C19677/A12834]. AG is partly supported by the NIHR Biomedical Research Centre, Oxford.

## Role of the funding source

The funder had no role in study design, in the collection, analysis, and interpretation of data, in the writing of the report, and in the decision to submit this article for publication.

## Declaration of competing interest

Hisham Mehanna reports personal fees from Warwickshire Head Neck Clinic Ltd, AstraZeneca, MSD, Sanofi Pasteur, and Merck; grants from GlaxoSmithKline Biologicals, MSD, Sanofi Pasteur, Silence Therapeutics, GlaxoSmithKline, AstraZeneca, and several academic funders including the National Institute for Health Research (NIHR) Health Technology Assessment Unit, Cancer Research UK, and the Medical Research Council; and travel expenses from Sanofi Pasteur, MSD, and Merck, outside the submitted work.

All other authors declare no conflict of interests.
