# Supplementary material for: Concurrent cisplatin or cetuximab with radiotherapy for HPV-positive oropharyngeal cancer: Medical resource use, costs, and quality-adjusted survival from the De-ESCALaTE HPV trial
Source: Eur J Cancer. 2020 Jan;124:178–85. doi: 10.1016/j.ejca.2019.10.025 (PMC6947474; doi:10.1016/j.ejca.2019.10.025)
Supplement: Multimedia component 1 [file mmc1.docx]

**Appendix**

**Medical resource use and costs**

**Treatment**

For patients in the cisplatin arm, centre-specific hydration and anti-emetic regimes were collated as part of another study. This allowed for detailed costing of items taken during each cycle of cisplatin. Additionally from the centre regimes, the length of time a patient received infusion could also calculated. Using clinical input, patients receiving infusions for 8 hours or more were classified as daycase and regular day/night cases, whilst those receiving infusions for less than 8 hours outpatients, and costed as such. Delivery of cetuximab is far simpler for the patient, requiring no hydration. Although no such analysis of centre regimes was performed, from communication with clinicians and a confirmatory look at a sample of regimes the most common drugs given alongside cetuximab were chlorphenamine (10mg) and dexamethasone (8mg), and, less frequently, ranitidine (150mg).

These items were costed using prices from the Department of Health and Social Care’s drugs and pharmaceutical electronic market information tool (eMIT)[1] and the British National Formulary (BNF).[2] The lowest costing product from the BNF was used when multiple products were available. For items used during the hospital visits for the chemotherapy cycle, pack sizes greater than one were converted into a cost per item. This was then applied to the number of items used. For oral medications taken at home, the cost of the whole pack was applied. Where fluid amounts given did not fully correspond with that of the reference item, wastage was assumed. Patients who did not receive their chemotherapy cycle were assumed to have not have medical resource use.

Finally, all trial patients received a dose of 65 Gy or more, and 99% received IMRT. Patients were therefore costed as having 35 fractions of IMRT.

**Impatient stays**

Treatment and follow-up case report forms (CRFs) contained information on the number of hospital admissions, their length and their reason (either as a pre-specified code or free text). Resource use questionnaires (RUQs) collected total number of inpatient overnight stays, which could be multiple admissions, with details given (when reported) in a free text section which was not specific to inpatient stays. To avoid double counting whilst maximising the use of available information, both the CRF and RUQ were used, with total overnight stays from the CRF taking precedence when both had positive usage as it was assumed to be more accurate.

Assigning costs to inpatient admissions was problematic given the dual sources. The CRF data provided greater granularity with the pre-specified reason codes Although going through free text from the CRFs was infeasible, in order to assign more accurate unit costs for these admissions common procedures for this patient group and the most common terms in the free text were coded for including: neck dissection, salvage surgery, PEG/RIG insertion, biopsy/panendoscopy, anorexia, and dehydration in order to assign more accurate unit costs. The pre-specified reasons and above admittance terms were mapped to OPCS-4 and ICD-10 codes using NHS Digital’s SNOMED CT Browser.[3] These code were then inputted into the NHS’s reference cost grouper to derive relevant HRG currency codes, from which unit costs could be obtained using the NHS national reference cost schedule.[4] An average cost of inpatient stay from the CRF data for each time point was calculate, and assigned to patients with stays only recorded in the RUQ.

**Day cases**

Day cases were also recorded in the CRFs as hospital admission with length of stays of zero. After exploring free text details in the RUQs and conversations with clinicians, it apparent that patients were recording day cases in both the day centre and outpatient visit questions. Day cases were thus manually created by searching the free text for procedures that were likely day cases – day surgery, tonsillectomy, biopsy and PEG/RIG insertion or removal. The corresponding days were then removed from outpatient/daycentre to avoid double counting. Costs were then assigned based on reason for admission

**Outpatient visit**

The RUQs was used as the sole source for outpatient visits. Although the follow-up CRFs contained information on some clinical assessments and imaging, allowing for greater granularity, there was no way of accurately separating this from the RUQs which recorded all activity. After the manual removal of day cases, the number of day centre visits was added the number of outpatient visits recorded. The weighted average of all outpatient attendances was then applied.

**Primary care and community-based services**

A large range of primary care and community-based services were asked for in the RUQs, including separation between face-to-face and telephone/email contacts, leading to over 30 variables. For many time points there were very few positive responses, posing a big problem to for our multiple imputation model. As such, questions were aggregated into the GP, District Nurse, Therapist, and Other variables. An average cost for these categories was calculated based on the complete data and assigned to imputed data points.

**Unit costs**

Table S1: Chemo/Bio-therapy ancillary drug costs

| **Hydration/anti-emetic** | **Item** | | **Source** | **Cost** | **Cost per item** |
| --- | --- | --- | --- | --- | --- |
| Hydration | Normal Saline (0.9% sodium chloride) | 2 litre | BNF[2] | £3.01 | £3.01 |
|  |  | 1 litre |  | £2.33 | £2.33 |
|  |  | 500ml |  | £1.61 | £1.61 |
|  |  | 100ml |  | £0.55 | £0.55 |
|  | KCL + Normal Saline (0.9% sodium chloride) | 1l + 40mmol | BNF[2] | £2.20 | £2.20 |
|  |  | 1l + 20mmol |  | £2.20 | £2.20 |
|  |  | 1l + 10mmol |  | £1.30 | £1.30 |
|  |  | 500ml + 20mmol KCl |  | £1.67 | £1.67 |
|  | KCL | 20mmol | BNF[2] | £2.66 | £0.27 |
|  | MgSO4 | 4mmol | eMIT[1] | £50.79 | £5.08 |
|  | Glucose | 5% 500ml | BNF[2] | £2.48 | £2.48 |
| Anti-emetic | Aprepitant | 125mg po | BNF[2] | £79.03 | £39.52 |
|  |  | 80mg po |  | £31.61 | £15.81 |
|  | Fosaprepitant | 150mg iv | BNF[2] | £47.42 | £47.42 |
|  | Ondansetron | 8mg iv | eMIT[1] | £0.58 | £0.12 |
|  |  | 8mg po |  | £0.75 | £0.08 |
|  |  | 4mg po |  | £1.49 | £0.15 |
|  | Dexamethasone | 6.6mg iv | eMIT[1] | £3.77 | £0.75 |
|  |  | 3.3mg iv |  | £2.39 | £0.48 |
|  |  | 2mg po |  | £14.46 | £0.29 |
|  | Lorazepam | 1mg po | eMIT[1] | £0.94 | £0.03 |
|  | Granisetron | 3mg iv | BNF[2] | £24.00 | £4.80 |
|  |  | 2mg po |  | £52.39 | £10.48 |
|  |  | 1mg po |  | £40.79 | £8.16 |
|  | Metoclopramide | 10mg po | BNF[2] | £0.45 | £0.02 |
|  | Cyclizine | 50mg po | eMIT[1] | £5.02 | £0.05 |
|  | Domperidone | 10mg po | eMIT[1] | £0.57 | £0.01 |
| Diuretics | Mannitol | 20% 500ml | BNF[2] | £6.46 | £6.46 |
|  |  | 10% 500ml |  | £4.92 | £4.92 |
|  | Furosemide | 20mg iv | eMIT[1] | £0.87 | £0.09 |
|  |  | 40mg po |  | £0.11 | £0.00 |
|  |  | 20mg po |  | £0.09 | £0.00 |
| Antihistimine | Chlorphenamine | 10mg iv | eMIT[1] | £10.44 | £2.09 |
|  | Ranitidine | 150mg | eMIT[1] | £0.66 | £0.01 |

Table S2: Chemo/Bio-radiotherapy and primary and social care costs

| **Item** | **Unit Cost** | **Source** | **Description** |
| --- | --- | --- | --- |
| Cisplatin | £10.13 | eMIT[1] | 2 X Cisplatin 100mg/100ml solution for infusion vials / Packsize 1 |
| Carboplatin | £28.24 | eMIT[1] | Carboplatin 600mg/60ml solution for infusion vials / Packsize 1 |
| Cetuximab | £890.50 | BNF[2] | Erbitux 500mg/100ml solution for infusion vials (Merck Serono Ltd) (2x for loading dose) |
| Delivery of Cis/Carboplatin and Cetuximab | £174  £375 (£252) | NHS reference costs 2017/18[4] | Cetuximab: Outpatient. Deliver Simple Parenteral Chemotherapy at First Attendance  Cis/Carboplatin: Daycase and Reg Day/Night (outpatient). Deliver Complex Chemotherapy, including Prolonged Infusional Treatment, at First Attendance |
| Radiotherapy (IMRT) | £135  £1546 | NHS reference costs 2017/18[4] | Deliver a Fraction of Complex Treatment on a Megavoltage Machine. HRG currency code: SC23Z (outpatient) (x)  Preparation for Intensity Modulated Radiation Therapy, with Technical Support. HRG currency code: SC41Z (outpatient) |
| Inpatient | See Supplementary Table 3 | | |
| Day case |  |  |  |
| Outpatient | £137 | PSSRU 2018[5] – based on NHS reference costs 2017/18[4] | Weighted average of all outpatient attendances |
| Accident & Emergency | £160 | NHS reference costs 2017/18[4] | Accident and Emergency Services, weighted average of all admitted codes. |
| Nursing/ Convalescent home | £152 | NHS reference costs 2017/18[4] | Specialist Palliative Care. Weighted average of all adult codes. |
| GP, surgery visit | Face to face: £37  Phone/email: £28 | PSSRU 2017[6] | p162, per surgery consultation lasting 9.22 minutes  Uses above estimate. Assuming telephone consultation lasts 7.1 minutes as suggest in PSSRU 2015 base on UK general practice workload survey |
| GP, home visit | Face to face:£46 | PSSRU 2017[6] | Uses above estimate. Assuming home consultation lasts 11.4 minutes as suggest in PSSRU 2015 base on UK general practice workload survey |
| District nurse, health visitor, community health team | Face to face: £37  Phone/email: £17 | NHS reference costs 2017/18[4] | District Nurse, Adult, Face to face. HRG currency code: N02AF  District Nurse, Adult, Non face to face. HRG currency code: N02AN |
| Social worker | Face to face: £82 | PSSRU 2017[6] | p174, £43 (£59) per hour; £59 (£82) per hour of client-related work. (Costs including qualifications given in brackets). Assuming one hour visit for face to face. |
| Physiotherapist | Face to face: £48  Phone/email: £41 | NHS reference costs 2017/18[4] | Weighted average of consultant led, non-consultant led Non-Admitted Face-to-Face Attendance, Follow-up (HRG currency code: WF01A) and Community health services Physiotherapist, Adult, One to One (HRG currency code: A08A1)  Weighted average of consultant led, non-consultant led Non-Admitted Non-Face-to-Face Attendance, Follow-up (HRG currency code: WF01C) |
| Occupational Therapist | Face to face: £70  Phone/email: £44 | NHS reference costs 2017/18[4] | Weighted average of consultant led, non-consultant led Non-Admitted Face-to-Face Attendance, Follow-up (HRG currency code: WF01A) and Community health services Occupational Therapist, Adult, One to One (HRG currency code: A06A1)  Weighted average of consultant led, non-consultant led Non-Admitted Non-Face-to-Face Attendance, Follow-up (HRG currency code: WF01C) |
| Counsellor | Face to face: £54  Phone/email: £34.72 | PSSRU 2017[6] | p180 (family support worker) , £32 per hour; £54 per hour of client-related work  From mean cost ratio of face to phone from other items |
| Home help or care worker | Face to face: £26  Phone/email: £16.72 | PSSRU 2017[6] | p178, Based on the price multipliers for independent sector home care provided for social services:  Face-to-face: £26 per hour weekday (£27 per day-time weekend, £27 per night-time weekday, £27 per night-time weekend).  From mean cost ratio of face to phone from other items |
| Citizens advice or welfare rights advisor | One appointment: £3.37  More than one: £170.62 | Citizens Advice Bureau[7] | £3 per client receiving Gateway advice (information, signposting and referral, usually a short appointment of 15-20 minutes).  £152 per client receiving full advice (detailed advice and ongoing case work, appointments normally last an hour, but many will require several appointments). 12/13 prices inflated to 17/18 using CPI[8] |
| Psychiatrist or psychologist | Face to face: £155  Phone/email: £80 | NHS reference costs 2017/18[4] | Weighted average of consultant led, non-consultant led Non-Admitted Face-to-Face Attendance, Follow-up (HRG currency code: WF01A)  Weighted average of consultant led, non-consultant led Non-Admitted Non-Face-to-Face Attendance, Follow-up (HRG currency code: WF01C) |
| Day centre | £92 | NHS reference costs 2017/18[4] | Day Care Facilities Regular Attendances, Other Patients. HRG currency code: DCF30 |
| Lunch or social club | £6.73 | Romeo et al[9] | £6, 09/10 prices inflated to 17/18 using CPI[8] |
| Food, medicine or laundry delivery service | £3.60 | National Association of Care Catering[10] |  |
| Family or patient support or self help groups | £43.35 | PSSRU 2010[11] | £0.57 per minute, assuming 1 hour, 09/10 prices inflated to 17/18 using CPI[8] |
| Other | £26.35 | Walsh et al 2017[12] | Cost of Macmillan Cancer Support nurse visit inflated to 17/18 using CPI[8] |

Table S3: Inpatient hospitalisation costs

To more accurately estimate the costs of hospitalisation, pre-specified hospital admission reasons were searched in the NHS [SNOWMED CT](https://termbrowser.nhs.uk/) browser which maps SNOMED terms into OPCS-4 and ICD-10 classifications. These codes were then mapped to HRG codes using HRG reference cost grouper 2017/18

| **Pre-specified hospital admission reason in case report forms** | **Unit cost** | **HRG Codes** | **HRG Description** | **Method** |
| --- | --- | --- | --- | --- |
| Myelosuppression | £1426 (£382) | SA08# | Other Haematological or Splenic Disorders. | Weighted average across EL and NEL (DC) admissions including all CC splits. |
| Sepsis/Infection/fever | £1968 (£370) | WJ06# | Sepsis | Weighted average across EL and NEL (DC) admissions including all intervention and CC splits. |
| CV toxicity | £1057  (£493) | EB03#  EB04Z  EB07#  EB10#  EB12#  EB13#  EB14# | Heart Failure or Shock  Hypertension  Arrhythmia  Actual or Suspected Myocardial Infarction  Unspecified Chest Pain  Angina  Other Acquired Cardiac Conditions | Weighted average across EL and NEL (DC) admissions including all intervention and CC splits. |
| GI toxicity/diarrhoea | £1371 (£374) | FD10# | Non-Malignant Gastrointestinal Tract Disorders | Weighted average across EL and NEL (DC) admissions including all intervention and CC splits. |
| Oral toxicity/mucositis | £797 (£397) | CB02# | Non-Malignant, Ear, Nose, Mouth, Throat or Neck Disorders | Weighted average across EL and NEL (DC) admissions including all intervention and CC splits. |
| Dyspnoea/cough | £597 (£528) | DZ19# | Other Respiratory Disorders | Weighted average across EL and NEL (DC) admissions including all intervention and CC splits. |
| Skin sensitivity/ulceration | £1463 (£434) | JD07# | Skin Disorders | Weighted average across EL and NEL (DC) admissions including all intervention and CC splits. |
| Anaphylactic reaction | £447 (£312) | WH05Z | Allergy or Adverse Allergic Reaction | Weighted average across EL and NEL (DC) admissions. |
| Other reasons (from free text match): |  |  |  |  |
| Neck dissection or salvage surgery | £4438 (£1587) | CA93# | Major Maxillofacial Procedures, 19 years and over | Weighted average across EL (DC) admissions including all CC splits. |
| Biopsy or panendoscopy | £1647 (£1008) | CA69A | Diagnostic, Laryngoscopy or Pharyngoscopy, 19 years and over | EL (DC) admissions. |
| PEG insertion | £1015 (£595) | FE12A | Endoscopic Insertion of Gastrostomy Tube, 19 years and over | EL (DC) admissions. |
| PEG removal | £607 | FE20Z | Therapeutic Endoscopic Upper Gastrointestinal Tract Procedures, 19 years and over | DC admission |
| RIG insertion | £1256 (£874) | YF01A | Radiological Insertion of Gastrostomy Tube, 19 years and over | EL (DC) admissions. |
| Dehydration | £1214  (£339) | KC05# | Fluid or Electrolyte Disorders | Weighted average across EL and NEL (DC) admissions including all intervention and CC splits. |
| Anorexia | £1657 (£368) | FD04# | Nutritional Disorders | Weighted average across EL and NEL (DC) admissions including all intervention and CC splits. |
| Tonsillectomy | £1425 | CA60C | Tonsillectomy, 4 years and over | DC admission |
| All other | £1931  (£775) |  |  | Weighted average across EL and NEL (DC) admissions including all intervention and CC splits. Excluding paediatrics. |

**Per protocol results**

Table S4: Medical resource use and costs

|  | **Cisplatin (n=166) Mean (SE)** | **Cetuximab (n=168) Mean (SE)** | **Mean difference (95% CI)** | **P-value** |
| --- | --- | --- | --- | --- |
| **Medical resource use** |  |  |  |  |
| **Treatment cycles** | 2.39 (SD: 0.59) | 7.55 (SD: 1.02) |  |  |
| **Hospital inpatient stays (total days)** | 10.172 (1.11) | 8.241 (1.079) | -1.931 (-4.977 to 1.115) | 0.213 |
| **Hospital day/outpatient visits** | 15.221 (1.105) | 15.487 (1.318) | 0.266 (-3.212 to 3.744) | 0.879 |
| **Accident and Emergency visits** | 0.385 (0.060) | 0.534 (0.083) | 0.148 (-0.052 to 0.349) | 0.147 |
| **Primary and community care contacts** | 24.592 (2.548) | 24.476 (2.135) | -0.116 (-6.524 to 6.292) | 0.972 |
| **Direct medical costs (£)** |  |  |  |  |
| **Treatment*** | 7322.619 (26.026) | 15183.719 (98.195) | 7861.100 (7659.594 to 8062.606) | 0.000 |
| **Hospital inpatient stays** | 2861.982 (41.678) | 2443.786 (229.185) | -418.194 (-1073.236 to 236.849) | 0.210 |
| **Hospital day/outpatient visits** | 2479.899 (148.711) | 2559.279 (179.695) | 79.380 (-376.035 to 534.795) | 0.731 |
| **Accident and Emergency visits** | 61.635 (9.606) | 85.383 (13.28) | 23.747 (-8.389 to 55.884) | 0.147 |
| **Primary and community care contacts** | 963.947 (107.305) | 911.607 (83.646) | -52.340 (-315.157 to 210.476) | 0.695 |
| **Total** | 13696.772 (347.742) | 21183.774 (368.129) | 7487.002 (6494.197 to 8479.806) | 0.000 |

*Including study drugs, other medications received during the cycle, delivery costs, and radiotherapy

Table S5: Unadjusted and adjusted cumulative mean quality-adjusted life-years (QALYs) from baseline

|  | **Cisplatin** | | **Cetuximab** | | **Mean difference (95% CI)** | **P-value t-test** |
| --- | --- | --- | --- | --- | --- | --- |
| **Time point** | **No. Dead** | **Mean (SE)** | **No. Dead** | **Mean (SE)** |  |  |
| ***4a: Unadjusted*** | | | | | | |
| **End of treatment** | 0 | 0.120 (0.002) | 1 | 0.114 (0.002) | -0.006 (-0.012 to 0.000) | 0.046 |
| **3 months after treatment end** | 1 | 0.295 (0.005) | 2 | 0.277 (0.005) | -0.017 (-0.033 to -0.002) | 0.026 |
| **6 months after treatment end** | 3 | 0.495 (0.008) | 5 | 0.465 (0.008) | -0.030 (-0.052 to -0.008) | 0.009 |
| **12 months after treatment end** | 4 | 0.905 (0.014) | 7 | 0.848 (0.016) | -0.057 (-0.098 to -0.016) | 0.006 |
| **24 months after treatment end** | 4 | 1.740 (0.027) | 16 | 1.612 (0.034) | -0.129 (-0.215 to -0.043) | 0.003 |
| ***4b: Adjusted*** | | | | | | |
| **End of treatment** |  | | | | -0.003 (-0.008 to 0.001) | 0.164 |
| **3 months after treatment end** |  |  |  |  | -0.012 (-0.025 to 0.001) | 0.080 |
| **6 months after treatment end** |  |  |  |  | -0.022 (-0.041 to -0.002) | 0.029 |
| **12 months after treatment end** |  |  |  |  | -0.044 (-0.081 to -0.007) | 0.021 |
| **24 months after treatment end** |  |  |  |  | -0.107 (-0.186 to -0.025) | 0.010 |

**Complete case analysis results**

Complete case analysis looks at the set of patients who had fully complete questionnaires at each time point (including dead patients). Of 334 patients, 119 were complete cases, 65 for cisplatin and 54 for cetuximab.

Table S6: Medical resource use and costs

|  | **Cisplatin (n=166) Mean (SE)** | **Cetuximab (n=168) Mean (SE)** | **Mean difference (95% CI)** | **P-value** |
| --- | --- | --- | --- | --- |
| **Medical resource use** |  |  |  |  |
| **Hospital inpatient stays (total days)** | 10.692 (1.643) | 8.888 (1.846) | -1.804 (-6.682to 3.075) | 0.465 |
| **Hospital day/outpatient visits** | 14.887 (1.712) | 13.376 (1.816) | -1.511 (-6.546 to 3.524) | 0.553 |
| **Accident and Emergency visits** | 0.418 (0.082) | 0.410 (0.100) | -0.008 (-0.265 to 0.250) | 0.954 |
| **Primary and community care contacts** | 22.021 (3.828) | 26.528 (4.261) | 4.507 (-6.840 to 15.854) | 0.433 |
| **Direct medical costs (£)** |  |  |  |  |
| **Treatment*** | 7042.602 (197.245) | 15076.626 (299.172) | 8034.025 (7344.645 to 8723.404) | 0.000 |
| **Hospital inpatient stays** | 3075.425 (428.693) | 2671.640 (457.715) | -403.785 (-1648.131 to 840.562) | 0.522 |
| **Hospital day/outpatient visits** | 2436.188 (247.603) | 2404.150 (255.819) | -32.038 (-743.585 to 679.508) | 0.929 |
| **Accident and Emergency visits** | 66.831 (13.167) | 65.630 (15.994) | -1.201 (-42.335 to 39.933) | 0.954 |
| **Primary and community care contacts** | 808.434 (147.250) | 969.570 (159.485) | 161.136 (-269.063 to 591.335) | 0.459 |
| **Total** | 13445.849 (616.382) | 21187.616 (703.532) | 7741.767 (5895.901 to 9587.634) | 0.000 |

*Including study drugs, other medications received during the cycle, delivery costs, and radiotherapy

Table S7: Unadjusted and adjusted cumulative mean quality-adjusted life-years (QALYs) from baseline

|  | **Cisplatin** | | **Cetuximab** | | **Mean difference (95% CI)** | **P-value t-test** |
| --- | --- | --- | --- | --- | --- | --- |
| **Time point** | **No. Dead** | **Mean (SE)** | **No. Dead** | **Mean (SE)** |  |  |
| ***4a: Unadjusted*** | | | | | | |
| **End of treatment** | **0** | 0.120 (0.003) | **0** | 0.115 (0.004) | -0.005 (-0.015 to 0.004) | 0.251 |
| **3 months after treatment end** | **0** | 0.297 (0.008) | **1** | 0.279 (0.009) | -0.019 (-0.042 to -0.005) | 0.115 |
| **6 months after treatment end** | **1** | 0.505 (0.011) | **1** | 0.470 (0.013) | -0.034 (-0.069 to 0.000) | 0.053 |
| **12 months after treatment end** | **1** | 0.931 (0.021) | **1** | 0.863 (0.026) | -0.068 (-0.133 to -0.003) | 0.040 |
| **24 months after treatment end** | **1** | 1.793 (0.041) | **3** | 1.658 (0.054) | -0.134 (-0.266 to -0.002) | 0.047 |
| ***4b: Adjusted*** | | | | | | |
| **End of treatment** |  | | | | -0.003 (-0.010 to 0.004) | 0.391 |
| **3 months after treatment end** |  |  |  |  | -0.014 (-0.034 to 0.006) | 0.184 |
| **6 months after treatment end** |  |  |  |  | -0.027 (-0.057 to 0.003) | 0.082 |
| **12 months after treatment end** |  |  |  |  | -0.056 (-0.114 to 0.002) | 0.058 |
| **24 months after treatment end** |  |  |  |  | -0.113 (-0.234 to 0.008) | 0.068 |

**Quality of life**

Table S8: Distribution of EQ-5D level response by domain

| **Time point** | **Response** | **Domain** | | | | | | | | | |
| --- | --- | --- | --- | --- | --- | --- | --- | --- | --- | --- | --- |
|  |  | **Mobility** | | **Self-care** | | **Usual activities** | | **Pain/discomfort** | | **Anxiety/depression** | |
|  |  | **Cisplatin** | **Cetuximab** | **Cisplatin** | **Cetuximab** | **Cisplatin** | **Cetuximab** | **Cisplatin** | **Cetuximab** | **Cisplatin** | **Cetuximab** |
| **Baseline** | **1** | 149 (96.1%) | 138 (90.2%) | 149 (96.1%) | 148 (96.7%) | 134 (86.5%) | 124 (81.0%) | 75 (48.4%) | 59 (38.6%) | 78 (50.3%) | 74 (48.7%) |
|  | **2** | 2 (1.3%) | 11 (7.2%) | 4 (2.6%) | 5 (3.3%) | 13 (8.4%) | 22 (14.4%) | 58 (37.4%) | 68 (44.4%) | 60 (38.7%) | 50 (32.9%) |
|  | **3** | 3 (1.9%) | 3 (2.0%) | 2 (1.3%) | 0 (0%) | 6 (3.9%) | 3 (2.0%) | 17 (11.0%) | 25 (16.3%) | 14 (9.0%) | 24 (15.8%) |
|  | **4** | 1 (0.7%) | 0 (0.0%) | 0 (0.0%) | 0 (0.0%) | 1 (0.6%) | 1 (0.6%) | 5 (3.2%) | 1 (0.7%) | 1 (0.6%) | 2 (1.3%) |
|  | **5** | 0 (0.0%) | 1 (0.6%) | 0 (0.0%) | 0 (0.0%) | 1 (0.6%) | 3 (2.0%) | 0 (0.0%) | 0 (0.0%) | 2 (1.3%) | 2 (1.3%) |
|  | **p value** | 0.030 | | 0.585 | | 0.289 | | 0.081 | | 0.405 | |
| **End of treatment** | **1** | 88 (71.0%) | 92 (67.6%) | 102 (82.3%) | 104 (76.5%) | 18 (14.6%) | 23 (16.9%) | 12 (9.7%) | 2 (1.5%) | 51 (41.5%) | 60 (44.1%) |
|  | **2** | 20 (16.1%) | 30 (22.1%) | 16 (12.9%) | 23 (16.9%) | 34 (27.6%) | 30 (20.1%) | 31 (25.0%) | 36 (26.5%) | 47 (38.2%) | 49 (36.0%) |
|  | **3** | 12 (9.7%) | 13 (9.6%) | 5 (4.0%) | 8 (5.9%) | 38 (30.9%) | 37 (27.2%) | 54 (43.5%) | 74 (54.4%) | 21 (17.1%) | 20 (14.7%) |
|  | **4** | 4 (3.2) | 1 (0.7%) | 1 (0.8%) | 1 (0.7%) | 13 (10.6%) | 14 (10.3%) | 25 (20.2%) | 17 (12.5%) | 2 (1.6%) | 6 (4.4%) |
|  | **5** | 0 (0.0%) | 0 (0.0%) | 0 (0.0%) | 0 (0.0%) | 20 (16.3%) | 32 (23.5%) | 2 (1.6%) | 7 (5.1%) | 2 91.6%) | 1 (0.7%) |
|  | **χ²** | 0.346 | | 0.751 | | 0.555 | | 0.005 | | 0.675 | |
| **3 Months after treatment end** | **1** | 102 (77.9%) | 106 (81.5%) | 120 (91.6%) | 124 (95.4%) | 60 (45.8%) | 58 (44.6%) | 51 (38.9%) | 28 (21.5%) | 68 (52.3%) | 75 (57.7%) |
|  | **2** | 19 (14.5%) | 15 (11.5%) | 9 (6.9%) | 1 (0.8%) | 46 (35.1%) | 41 (31.5%) | 55 (42.0%) | 67 (51.5%) | 47 (36.2%) | 36 (27.7%) |
|  | **3** | 9 (6.9%) | 5 (3.8%) | 2 (1.5%) | 5 (3.8%) | 21 (16.0%) | 23 (17.7%) | 25 (19.1%) | 28 (21.5%) | 14 (10.8%) | 15 (11.5%) |
|  | **4** | 1 (0.8%) | 3 (2.3%) | 0 (0.0%) | 0 (0.0%) | 2 (1.5%) | 4 (3.1%) | 0 (0.0%) | 7 (5.4%) | 1 (0.8%) | 4 4 (3.1%) |
|  | **5** | 0 (0.0%) | 1 (0.8%) | 0 (0.0%) | 0 (0.0%) | 2 (1.5%) | 4 (3.1%) | 0 (0.0%) | 0 (0.0%) | 0 (0.0%) | 0 (0.0%) |
|  | **p value** | 0.442 | | 0.020 | | 0.792 | | 0.001 | | 0.319 | |
| **6 Months after treatment end** | **1** | 101 (78.9%) | 98 (78.4%) | 119 (93.0%) | 114 (91.2%) | 72 (56.3%) | 59 (47.2%) | 55 (43.0%) | 46 (36.8%) | 86 (67.2%) | 75 (60.0%) |
|  | **2** | 16 (12.5%) | 20 (16.0%) | 5 (3.9%) | 10 (8.0%) | 37 (28.9%) | 43 (34.4%) | 53 (41.4%) | 55 (44.0%) | 32 (25.0%) | 34 (27.2%) |
|  | **3** | 8 (6.3%) | 6 (4.8%) | 4 (3.1%) | 1 (0.8%) | 16 (12.5%) | 16 (12.8%) | 19 (14.8%) | 20 (16.0%) | 9 (7.0%) | 11 (8.8%) |
|  | **4** | 3 (2.3%) | 1 (0.8%) | 0 (0.0%) | 0 (0.0%) | 3 (2.3%) | 5 (4.0%) | 1 (0.8%) | 3 (2.4%) | 0 (0%) | 5 (4.0%) |
|  | **5** | 0 (0.0%) | 0 (0.0%) | 0 (0.0%) | 0 (0.0%) | 0 (0.0%) | 2 (1.6%) | 0 (0.0%) | 1 (0.8%) | 1 (0.8%) | 0 (0.0%) |
|  | **p value** | 0.651 | | 0.202 | | 0.423 | | 0.621 | | 0.113 | |
| **12 months after treatment end** | **1** | 109 (83.8%) | 107 (84.3%) | 124 (95.4%) | 120 (94.5%) | 94 (72.3%) | 82 (64.6%) | 70 (53.8%) | 62 (48.8%) | 89 (68.5%) | 82 (65.1%) |
|  | **2** | 17 (13.1%) | 11 (8.7%) | 4 (3.1%) | 4 (3.1%) | 25 (19.2%) | 29 (22.8%) | 46 (35.4%) | 45 (35.4%) | 30 (23.1%) | 31 (24.6%) |
|  | **3** | 2 (1.5%) | 8 (6.3%) | 2 (1.5%) | 3 (2.4%) | 9 (6.9%) | 10 (7.9%) | 14 (10.8%) | 13 (10.2%) | 9 (6.9%) | 10 (7.9%) |
|  | **4** | 2 (1.5%) | 1 (0.8%) | 0 (0%) | 0 (0%) | 2 (1.5%) | 6 (4.7%) | 0 (0%) | 4 (3.1%) | 2 (1.5%) | 3 (2.4%) |
|  | **5** | 0 (0%) | 0 (0%) | 0 (0%) | 0 (0%) | 0 (0%) | 0 (0%) | 0 (0%) | 3 (2.4%) | 0 (0%) | 0 (0%) |
|  | **p value** | 0.137 | | 0.912 | | 0.383 | | 0.122 | | 0.921 | |
| **24 months after treatment end** | **1** | 105 (87.5%) | 101 (85.6%) | 113 (94.2%) | 111 (94.1%) | 92 (76.7%) | 83 (70.3%) | 70 (58.3%) | 53 (44.9%) | 80 (66.7%) | 81 (68.6%) |
|  | **2** | 10 (8.3%) | 12 (10.2%) | 6 (5.0%) | 6 (5.1%) | 19 (15.8%) | 24 (20.3%) | 37 (30.8%) | 45 (38.1%) | 27 (22.5%) | 25 (21.2%) |
|  | **3** | 4 (3.3%) | 4 (3.4%) | 1 (0.8%) | 1 (0.8%) | 9 (7.5%) | 10 (8.5%) | 11 (9.2%) | 19 (16.1%) | 12 (1.0%) | 11 (9.3%) |
|  | **4** | 1 (0.8%) | 1 (0.8%) | 0 (0%) | 0 (0%) | 0 (0%) | 1 (0.8%) | 2 (1.7%) | 1 (0.8%) | 1 (0.8%) | 1 (0.8%) |
|  | **5** | 0 (0%) | 0 (0%) | 0 (0%) | 0 (0%) | 0 (0%) | 0 (0%) | 0 (0%) | 0 (0%) | 0 (0%) | 0 (0%) |
|  | **p value** | 0.953 | | 1.000 | | 0.592 | | 0.113 | | 0.979 | |

**References**

[1] Department of Health and Social Care. Drugs and Pharmaceutical Electronic Market Information Tool (eMit). 2018.

[2] Joint Formulary Committee. British National Formulary (online). <https://www.medicinescomplete.com> (accessed 04 November 2018).

[3] NHS Digital. SNOMED CT Browser. <https://termbrowser.nhs.uk/>.

[4] Department of Health and Social Care. Reference Costs 2017–2018. London2018.

[5] Curtis LA, Burns A. Unit Costs of Health and Social Care 2018. Canterbury: Personal Social Services Research Unit, University of Kent; 2018.

[6] Curtis LA, Burns A. Unit Costs of Health and Social Care 2017. Canterbury: Personal Social Services Research Unit, University of Kent; 2017.

[7] Citizens Advice Bureau. The value to society of the Citizens Advice service. 2014.

[8] Bank of England. Inflation calculator. <https://www.bankofengland.co.uk/monetary-policy/inflation/inflation-calculator>.

[9] Romeo R, Knapp M, Hellier J, Dewey M, Ballard C, Baldwin R, et al. Cost-effectiveness analyses for mirtazapine and sertraline in dementia: randomised controlled trial. The British Journal of Psychiatry. 2013;202:121-8. <https://doi.org/10.1192/bjp.bp.112.115212>

[10] National Association of Care Catering. Meals on Wheels Survey 2018. 2018.

[11] Curtis LA, Burns A. Unit Costs of Health and Social Care 2010. Canterbury: Personal Social Services Research Unit, University of Kent; 2010.

[12] Walsh TS, Stanworth S, Boyd J, Hope D, Hemmatapour S, Burrows H, et al. The Age of BLood Evaluation (ABLE) randomised controlled trial: description of the UK-funded arm of the international trial, the UK cost-utility analysis and secondary analyses exploring factors associated with health-related quality of life and health-care costs during the 12-month follow-up. Health Technology Assessment. 2017;21. <https://doi.org/10.3310/hta21620>
